# Efficacy and Safety of Different Dosage of Recombinant Tissue-type Plasminogen Activator (rt-PA) in the Treatment of Acute Pulmonary Embolism: A Systematic Review and Meta-analysis

**DOI:** 10.22037/ijpr.2021.114142.14688

**Published:** 2021

**Authors:** Shahideh Amini, Hooman Bakhshandeh, Reza Mosaed, Hamidreza Abtahi, Kourosh Sadeghi, Mojtaba Mojtahedzadeh

**Affiliations:** a *Department of Clinical Pharmacy, Faculty of Pharmacy, Tehran University of Medical Sciences, Tehran, Iran. *; b *Research Center for Rational Use of Drugs, Tehran University of Medical Sciences, Tehran, Iran. *; c *Rajaie Cardiovascular Medical and Research Center, Iran University of Medical Sciences, Tehran, Iran. *; d *Faculty of Medicine, Aja University of Medical Sciences, Tehran, Iran. *; e *Advanced Thoracic Research Center, Tehran University of Medical Sciences, Tehran, Iran.*

**Keywords:** Recombinant tissue-type Plasminogen Activator, rt-PA, Pulmonary embolism, Bleeding, Mortality, Recurrence

## Abstract

Reperfusion therapies are recommended for patients with hemodynamic instability or high-risk acute pulmonary embolism (PE). Lower doses of tissue plasminogen activator (rt-PA) could be considered to improve bleeding complications. The aim of this study was to evaluate the efficacy and safety of a reduced dose of rt-PA for the treatment of acute PE, compared with anticoagulation and standard dose. PubMed Central, Scopus, Web of Science and Embase were searched for all relevant randomized studies and prospective observational studies that compared reduced dose of rt-PA with anticoagulation alone or standard dose of rt-PA in patients with acute PE. The risk ratios (RR, with 95% CI) were calculated according to the value of I2. Outcomes were described as bleeding events, all-cause death, and recurrence of PE. Thirteen articles, including four observational studies (4223 patients) and nine RCTs (780 patients), were included. In comparing reduced dose of rt-PA with anticoagulant, a greater incidence of total bleeding events in low dose was showed (RR, 5.08 (95% CI, (1.39–18.6), I2 = 0.0%). In the standard dose rt-PA *vs.* reduced dose, there was a greater incidence of total bleeding events in the standard dose of rt-PA, RR 1.48 (95% CI, (1.00–2.19), I2 = 0.0%) was shown. There were no statistical differences in recurrent PE or all-cause mortality. It concluded that in the absence of the benefit of a standard dose of rt-PA in comparison with dose reduction, a reduced dose of rt-PA showed a lower rate of total bleeding events and similar efficacy regarding mortality and PE recurrence rate.

## Introduction

Acute Pulmonary embolism (PE) is a common and potentially fatal condition that can result in substantial morbidity and mortality. PE mortality rate increases exponentially with the age of the patient. PE contributes to significant fatality rates in intermediate-risk or submassive PE and also in high-risk or massive PE, as much as 3%–8% and 25%–52%, respectively (1-3).

In addition to the early consequence of PE, post-PE impairment remains a main late complication of acute PE that is associated with reduced functional capacity and increased health care expenditures (4, 5).

Reperfusion therapies are recommended by international guidelines in patients with hemodynamic instability or high-risk PE (6, 7). Current evidence shows that using thrombolytic in high-risk patients with PE can reduce mortality and improve recurrent PE outcomes; although, it is usually accompanied by the increased risk of major, intracranial and fatal bleeding. It seems that the benefit versus risk ratio of thrombolytic therapies is quite narrow and needs further investigations. (8). There is a debate about using a reduced dose of thrombolytic along with its benefit-risk balance in patients with acute PE and if it has a beneficial effect on clinical outcomes in comparison with the standard recommended dose.

Nevertheless, a number of previous trials ran on patients with acute PE demonstrated some clinical improvement by a reduction in dosage of systemic thrombolysis compared to anticoagulation in an attempt to improve the safety of thrombolytic therapy (9-11). A former review has concluded that a low-dose recombinant tissue plasminogen activator (RTPA) can be an effective and safe treatment option for acute PE, particularly in patients at greatest risk of bleeding (12). One other systematic review and meta-analysis revealed that low-dose RTPA had the same therapeutic effect and lowered bleeding risk compared with the standard dose of RTPA (13).

Hence, we conducted a systematic review of available literature to appraise the efficacy and safety of a reduced dose of RTPA for the management of acute PE in comparison to anticoagulation and the standard dose of RTPA, which is 100 mg.

## Experimental

*Methods*

*Sources of Data and Search strategy*

Methodology and the manuscript of the present study were organized as stated by the PRISMA statement and MOOSE guideline. The registration number in PROSPERO is CRD42020150370.

Searching in electronic databases consists of PubMed Central, Scopus, Web of Science and Embase was looking for all relevant published articles until April 30, 2019, without time restriction. Exclusively, we considered articles that were published in English. Medical subject headings (MeSH) and free text words were used to recognize the related potential evidence. In addition, the bibliography of retrieved studies was used to find further relevant articles. We researched the bibliographies of the retrieved articles manually to recognize additional unpublished and gray literature. 

The search strategy in PubMed Central, Scopus, Web of Science, and Embase was shown in Appendix 1 to 4 in supplementary file.

All articles, including randomized control trials (RCTs) and observational prospective cohort studies, were investigated by reviewing titles, abstracts and their methodological design (including sample size, population and design of the study) to determine appropriate references for eligibility criteria. EndNote X5 for Windows (Thomson Reuters, Philadelphia, Pennsylvania) was applied to manage the retrieved articles in order to merge different searches to remove duplications and also accelerate the review process.

*Study Selection*

Inclusion criteria for including studies were as following: 

(a) Study design and intervention of all clinical RCTs and observational prospective cohort studies were evaluated that compared: 1) a low dose (0.6 mg/kg or 50 mg) RTPA or a standard dose (100 mg) of RTPA versus unfractionated heparin (UFH), or low molecular weight heparin (LMWH); 2) or evaluating a low dose of RTPA ( maximum 50 mg or 0.6 mg/kg) versus a standard dose of RTPA (100 mg), and (b) if the outcomes of interest were mortality, PE recurrence, and hemorrhage adverse effect. Major bleeding was referred to fatal bleeding, such as intracranial hemorrhage (ICH) or hemoglobin concentration decline ≥ 2 g/dL, indicating the need for blood transfusion.

 We excluded non-English articles, letters, editorials, narrative reviews, case series and case reports. 

According to titles and abstracts, two investigators (Sh.A. and R.M.) conducted the comprehensive literature search, omitted duplicates, and selected studies. Two independent reviewers (Sh.A. and K.S.) screened the primary selections to access eligibility criteria. In a disagreement between the reviewers, they reached a consensus via discussion and consultation with a third reviewer (H.A.).

*Data Extraction*

Two authors (Sh.A. and R.M.) collected the following data by using a data extraction form independently.

 1) Study characteristics (author’s name, publication date [year], study design, sample size by study groups, type of thrombolytic, type of anticoagulant, dosage and administration type and follow-up duration).

2) Characteristics of the patients (study population, age, gender).

3) Study endpoints (frequencies and risk ratios for main outcomes in intervention and control groups) any disagreements were settled through discussion among authors during data extraction.

*Quality assessment*

Potential bias was independently evaluated by two reviewers (SH.A and H.B). Regarding the evaluation of clinical trials, Cochrane’s risk of bias (RoB2) tool, and for cohort studies, the Newcastle-Ottawa assessment scale (NOS) were applied. 

*Statistical Analysis*

Statistical analysis was conducted by using the Stata 13 for Windows (Stata Inc., TX, and USA). Pooled estimates for the study endpoints were presented as relative risks and their 95% confidence intervals [RR (CI95%)]. Each outcome was tested for heterogeneity. In the studies, heterogeneity was performed by using Cochrane’s Q test (significant with a *P*-value of < 0.10), and the I^2^ statistic indicated a relative amount of variance of the summary effect. If heterogeneity was presented, a random-effects model was applied (DerSimonian and Laird [Mantel-Haenszel model). To find the possibility of, heterogeneity, meta-regression models were applied. The existence of publication bias was assessed via funnel plots. The symmetry of funnel plots (small study effects) was investigated by the regression-based Egger test. *P*-value < 0.05 was defined as a statistically significant result. 

## Results

**Figure 1:**
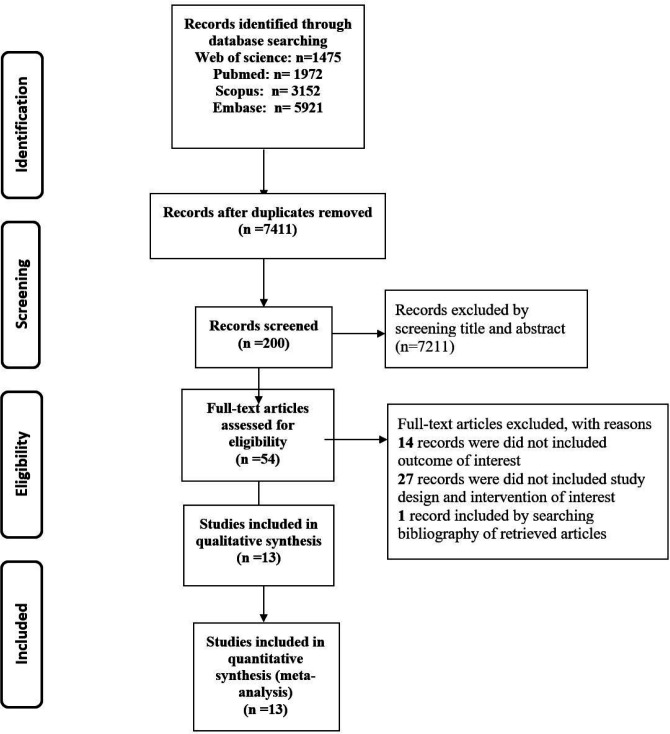
Study flow diagram

After screening 12,520 records, thirteen articles including four observational studies and nine clinical RCTs (n=780) were included (Figure 1). The summary of included studies is shown in Table 1. All entire articles have been published from 1990 to 2018. The follow-up duration varied from 7 days to 28 months.

*Study Selection*

The flow diagram of the search process is shown in Figure 1. The initial search identified 12,520 articles through PubMed Central, Scopus, Web of Science, and Embase. Among those publications, overall, 5,109 records were duplicates. After excluding duplicates, a number of 7,211 studies were retrieved for further screening by title and abstract evaluation. After that, 200 full-text articles were assessed for eligibility. The cited references of the included articles were analyzed to evaluated potential eligible studies; accordingly, one other related article was found by searching backward for references cited in those articles which had been found. Finally, 53 articles remained to full-text review. Out of 53 recognized papers, 27 articles were left out due to insufficient data, not having a control group, an intervention group, and different thrombolytic drugs (all of them considered a reason for “did not include study design and intervention of interest”). Fourteen records did not meet our inclusion outcome criteria and finally, 13 studies were left to include in our meta-analysis.

*Study Characteristics*

As shown in Table 1, the population of studies was different. 

Two observational studies (2, 14) compared a reduced-dose (0.6 mg/kg, utmost 50 mg) RTPA with a full dose of 100 mg. Two other observational studies (15, 16) compared a reduced dose of RTPA against an anticoagulant alone (heparin or enoxaparin). As expected in one study (2), the method of administration of RTPA in other observational cohort studies was a 2-hour infusion. 

Three clinical RCTs (10, 17 and 18) were compared to a reduced dose (0.6 mg/kg, utmost 50 mg) RTPA and an anticoagulant, three clinical RCTs (19-21) were compared to the dose of 100 mg RTPA and an anticoagulant, and three other clinical RCTs (11, 22 and 23) were compared 100 mg of RTPA with a reduced dose.

*Risk of Bias Assessment*

**Figure 2:**
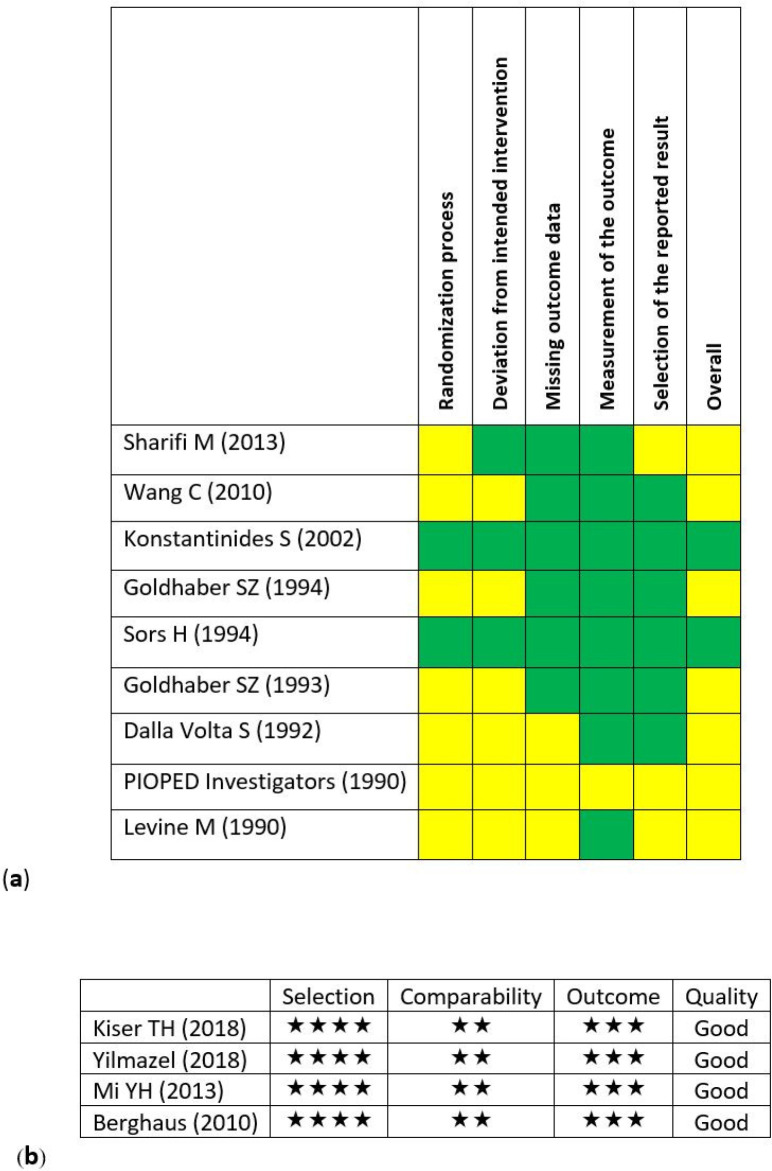
Assessment of the risk of bias in the primary studies. (a) Clinical trials (by using Cochran’s risk of bias (RoB2) tool). (Green: low risk, yellow: some concerns and red: high risk) (b) Cohort studies (by using Newcastle-Ottawa assessment scale (NOS)).

Nine interventional RCTs and 4 observational historical cohort studies were included in the final analysis. The findings of risk of bias assessment were presented in Figure 2. Within the clinical RCTs, 7 studies had shortages to some extent (Figure 2a). This may have occurred due to improper writing and not considering the standards of reporting the clinical RCTs. All 4 cohort studies were evaluated as good-quality studies (Figure 2b).

*Outcomes*

*Total Bleeding*

This study showed a greater occurrence of total bleeding (overall major and minor bleeding) in RTPAs rather than in the anticoagulant alone.

The pooled estimate of relative risk (RR) was assessed among subgroups based on the comparable drugs. In the subgroups of a reduced dose of RTPA and an anticoagulant, the greater incidence of total bleeding events in low-dose RTPA, RR was 5.08 (95% CI, (1.39–18.6), I^2 ^ = 0.0%)

In the subgroups of a standard dose of RTPA versus a reduced dose of RTPA, a greater incidence of total bleeding complications occurred in the standard dose of RTPA and RR was 1.48 (95% CI, (1.00–2.19), I^2 ^ = 0.0%). In other words, the incidence of bleeding was about 1.5 times greater in RTPA with the standard dose.

**Figure 3:**
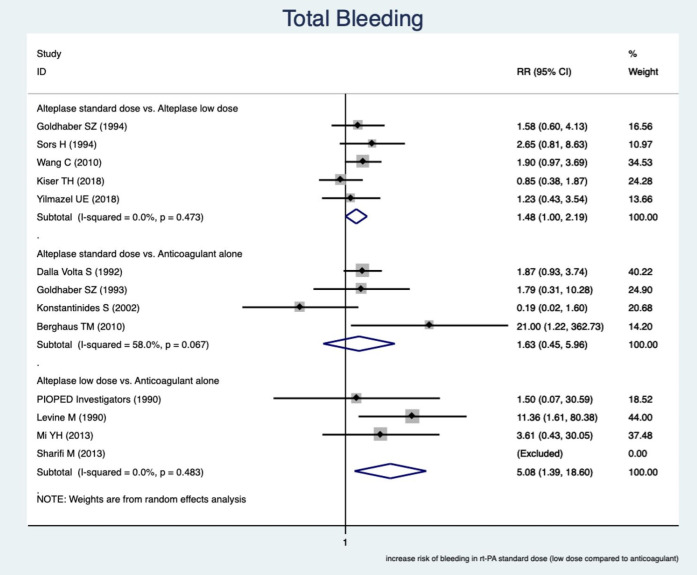
Forest plot of pooled estimate relative risk (RR) to evaluate the association between different subgroups and bleeding

In the subgroups of standard-dose RTPA against an anticoagulant, there was no significant difference in total bleeding events and RR was 1.63 (95% CI, (0.45 – 5.96), I^2 ^ = 58%). The unexpected findings in this subgroup were mainly because of the low precision study of Berghaus TM (2010), which affected the pooled estimate of RR. The findings are presented in Figure 3.

*Recurrent PE*

It showed that no significant difference in recurrent PE.

In the subgroups of a standard dose of RTPA versus a reduced dose, RR was 2.77 (95% CI, (0.84–9.16), I^2 ^ = 0.0%).

**Figure 4:**
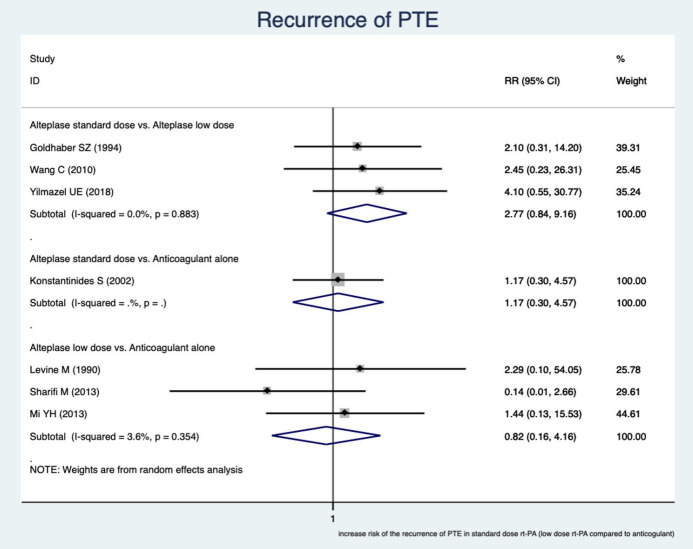
Forest plot of pooled estimate relative risk (RR) to evaluate the association between different subgroups and recurrent pulmonary embolism

In the subgroups of a reduced dose of RTPA counter to an anticoagulant, RR was 0.82 (95% CI, (0.16–4.16), I^2 ^ = 3.6%). The findings are presented in Figure 4.

*Mortality*

**Figure 5:**
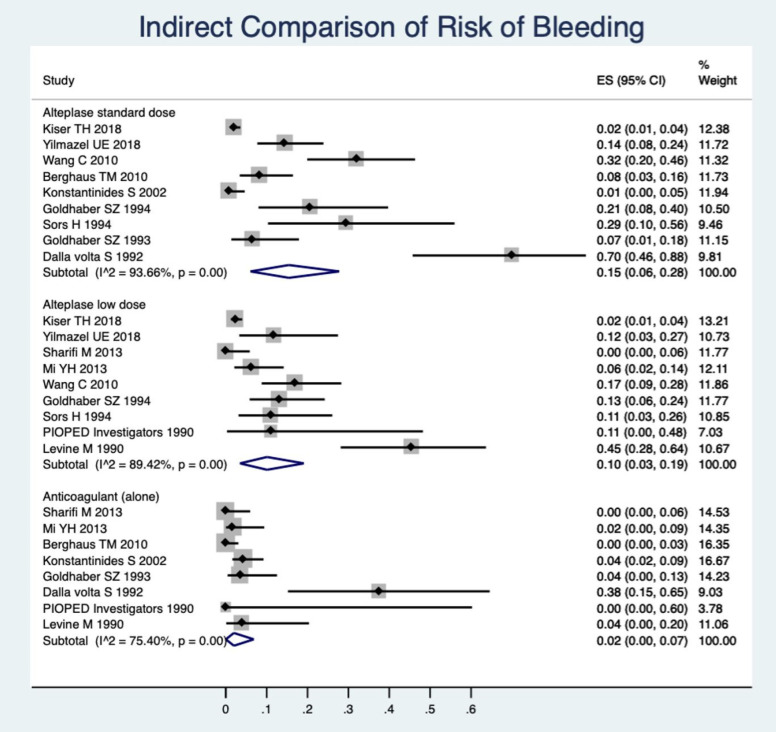
Indirect comparison of the pooled estimate of incidence rates of total bleeding between the anticoagulation drugs

Regarding all-cause mortality, no significant difference was seen. The findings are presented in Figure 5. 

*Indirect Comparisons*

As a complementary analysis, we made a descriptive meta-analysis to obtain pooled estimates of incidences of bleeding, mortality, and recurrence of PE among each type of medication. The pooled estimate of the occurrence of total bleeding was calculated within the single-arm trial of a standard dose of RTPA among the studies that had a single arm of the standard dose of RTPA. Similarly, pooled estimates for the incidence of total bleeding were calculated within the single arm of low dose RTPA and a single-arm anticoagulant among the relevant studies. The findings can be compared according to CI95% of the incidence rate. However, this is not an alternative for head-to-head comparisons; due to the great numbers of studies in a single arm, it can provide complementary information for interpretation of the previous findings. These results are presented in Figure 5. 

In Figure 5 the occurrence of bleeding is shown to be greater in the standard-dose rt-PA. Low-dose RTPA has an important lower incidence of bleeding events than the standard dose and shows the anticoagulant, alone, also has a considerably lower bleeding rate. 

*Sensitivity Analysis*

In order to consider the differences between the study types, pooled RRs were calculated in clinical RCTs and observational cohort studies. The results are presented in Table 2. We could not obtain the pooled estimate on several occasions because of the relatively lower number of cohort studies. This also happened for the clinical RCTs. Other pooled estimates were calculated by using 2 or more study results and, therefore, estimate precisions are low. This analysis increased the heterogeneity in virtually all estimations and compared the results presented above (Tables 2). The results are precise and applicable when the pooled estimation of RRs is achieved by bringing the results of both the clinical RCTs and cohort studies together. 

*Assessment of the Heterogeneity*

According to the findings of this study, heterogeneity can be found in the estimation of bleeding. We assessed the probable role of several factors to create such heterogeneity by using the meta-regression method. These factors included: Year of the conduction of primary studies, mean age of the participants, type of studies (cohort or RCT), instability of hemodynamic condition of study population and bolus administration of RTPA. The results suggested that earlier studies (*P*-value = 0.054) and cohort types (*P*-value = 0.056) were the factors associated with heterogeneity. The lower age of the study population can also be considered important (*P*-value = 0.091).

*Publication bias and effect of small studies*

**Figure 6:**
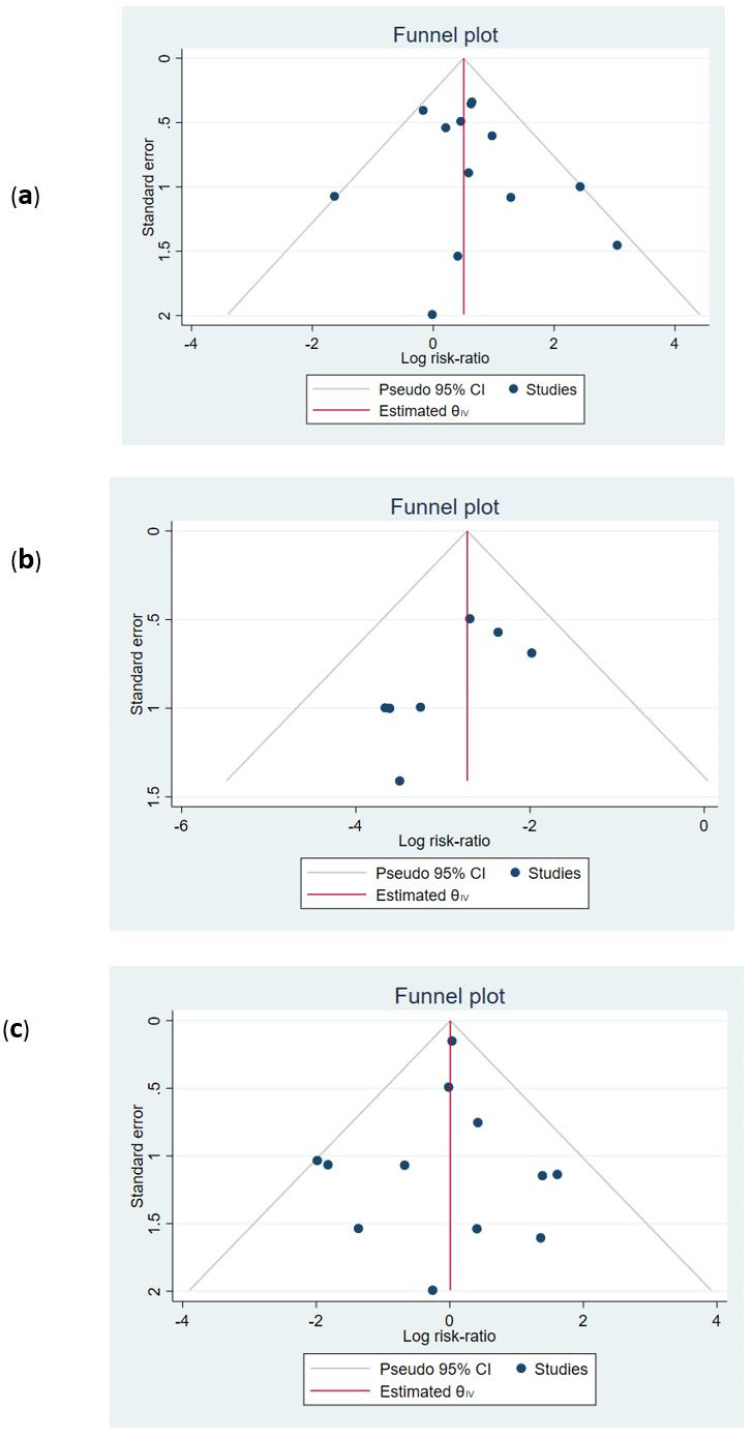
Funnel plot for the assessment of publication bias. (a) total bleeding; *p*-value for Egger test: 0.417. (b) Recurrence of pulmonary thrombo-emboli; Egger test *p*-value: 0.175. (c) Mortality; Egger test *p*-value: 0.720

Funnel plots were used to assess the existence of publication bias. The findings were presented in Figure 6. The symmetry of funnel plots (small study effects) was investigated by the regression-based Egger test. The *P*-value for the test showed that no asymmetry was found in the funnel plots. Hence, the probability of publication bias is low (all *P-*values > 0.05, Figures 6a-6c). 

## Discussion

According to international guidelines, thrombolytic rescue therapy is recommended for patients who deteriorate hemodynamically or patients with high-risk PE (6, 7). Previous studies showed that pulmonary obstruction, pulmonary atrial pressure (PAP), and pulmonary vascular resistance (PVR) in patients with PE, who received fibrinolysis, improved faster in comparison to heparin alone. Moreover, along with PVR reduction, an improvement in right ventricular (RV) dilation was seen (20, 21). It remains unclear whether thrombolysis for patients without hemodynamic decompensation (intermediate risk of PE or normotensive patients with RV dysfunction and troponin levels elevation) has a beneficial effect on the clinical outcome in comparison with the safety factor. However, the risk of bleeding events has remained an important limiting issue in the use of thrombolytic medication in clinical practice. There is a hypothesis that bleeding of RTPA is related to the amount of the dose (24).

In the current review, we came to the conclusion that 3 key findings regarding different doses of RTPA are apparent. First, findings from RCTs and observational studies in both safety and efficacy outcomes were consistent, by and large. The results were precise and applicable when the pooled estimation of RRs was achieved by bringing the results of both the clinical RCTs and cohort studies together. Second, the pooled results showed that the risk association of bleeding varied among different doses of RTPA as well as the incidence of bleeding was about 1.5 times greater in standard dose RTPA. It is well understood that the dose-dependent bleeding had occurred. Third, polled results in direct comparison to each other revealed that the rate of major bleedings was much lower than minor bleedings and the incidence of minor bleedings was similar in the standard and low dose of RTPA, and both were higher than the anticoagulant. 

To the best of our knowledge, Zhang *et al.* study (13) was the only systematic review before our study which investigated this subject. They evaluated the efficacy and safety of low-dose RTPA in the management of acute PE, compared with heparin and the standard dose of RTPA. They found that no significant difference in efficacy between low dose RTPA and a standard dose, but it showed fewer bleedings than a standard dose. Furthermore, in comparison with heparin, a reduced dose of RTPA did not magnify the risk of major bleedings (13).

Although this study provided suggestions for the PE treatment by reducing the dose of RTPA, interpretation of the study results appears to be limited because of the low number of included studies and patients. In addition, there was no conclusion about different types of PE, such as hemodynamic unstable or intermediate risk.

In line with former studies, we did not find any significant difference in the rate of recurrent PE or in all-cause mortality in the reduced dose of RTPA versus an anticoagulant alone or in reduced dose against the full dose of 100 mg. Nevertheless, total bleeding events occurred more frequently in the standard dose in comparison with the low-dose RTPA group. Moreover, we found the incidence of major bleeding to be similar in low dose RTPA and the anticoagulant, and the incidence of minor bleeding was similar in standard and low dose RTPA. According to the high number of patients in a recent study; thirteen studies, including four observational studies (4223 patients) and nine RCT (780 patients) that were analyzed, our findings were generally in line with the previous systematic review (13), although the large number in our study was seen as a strength property of this review.

Since a reduced dose of RTPA is not officially approved, this study, which included more patients than all other reviews, showed an absence of more efficacy benefit in mortality and PE recurrence using a standard dose of RTPA versus a reduced dose occurred. It also showed the rate of bleeding adverse events was lower with a reduced dose. However, regarding the risk-benefit ratio for thrombolysis treatment, a dose of 0.6 mg/kg within 15 min (utmost 50 mg) may be warranted in patients with indications for administrating thrombolytic therapy regardless of PE severity type.

**Table 1:** Main characteristics of the retrieved studies

**Study **	**Study design**	**Number of participants**	**Population**	**Intervention**	**Follow up**	**Thrombolytic administration**	**finding**
Kiser TH *et al*., 2018 (Ref 2)	cohort	3768	PTE^a^ without mention of risk category	Compare 50 mg RTPA^b ^versus full-dose 100 mg	No mention	No mention	Same efficacy, hospital mortality and major bleeding; however, patients receiving half-dose of RTPA were more likely to need further escalation in care for the management of their acute PTE.
Yilmazel UE *et al*., 2018 (ref: 14)	cohort	117	Massive and Submassive ^c^ (data analysis without mention of PTE risk category	Compare 50 mg of RTPA versus full-dose (100 mg)	3 months	50 mg administrated within 2 hours, 100 mg infused as intravenous bolus of 10 mg, followed by 90 mg within 2 h	Same benefit, hospital mortality and major bleeding.
Sharifi M *et al.*, 2013 (ref:^17^)	RCT ^d^	121	Moderate ^e^ PTE	Compare reduced dose of RTPA versus anticoagulant alone (enoxaparin)	28 months	Bolus of 10 mg within 1 minute followed by 40 mg infused over 2 h. if body weight <50 kg, a total dose of 0.5 mg/kg , was administrated as a bolus of 10-mg followed by the remainder over 2 h	Reduced dose of RTPA showed efficacy in care of patients with PE with a considerable decline in the pulmonary artery pressure that occurred within follow up of 28 months.
Mi YH *et al*., 2013 (ref: 15)	cohort	136	Submissive pulmonary embolism^f^	Compare reduced dose of RTPA versus anticoagulant alone (heparin or enoxaparin)	12 months	50 mg RTPA administrated over 2 h	Short- and long-term an outcome was more improved in RTPA reduced dose compared with enoxaparin without increasing major bleeding events. Reduced dose showed more improvement in short and long term *vs*. enoxaparin without excess major bleedings
Wang C *et al.*, 2010 (ref: 22)	RCT	118	Hemodynamic instability, Massive PTE^ g^	Compare reduced dose of rt-PA, 50 mg versus full-dose 100 mg	14 days	50 mg and 100 mg of RTPA administrated within 2 h	The dose of 50 mg showed similar efficacy, lower total bleedings, and mortality in compare with the dose of 100 mg.
Berghaus TM *et al.*, 2010 (ref: 16)	cohort	202	Intermediate^ h^PTE	Compare 100 mg of RTPA versus anticoagulant alone		100 mg of RTPA administrated within 2 h	More minor bleedings, same major bleeding and efficacy with shorter hospital stay was seen with RTPA
Konstantinides S *et al.*, 2002 (ref: 19)	RCT	256	Intermediate PTE	Compare 100 mg of RTPA versus anticoagulant alone (heparin)	30 days	100 mg of RTPA administrated within 2 h	Both groups showed very low rate of death and bleedings. Clinical course improvement was seen with RTPA.
Goldhaber SZ *et al.*, 1994 (ref: 11)	RCT	90	Hemodynamically stable	Compare reduced dose of 50 mg versus 100 mg of RTPA	14 days	50 mg was given over 15 min and 100 mg of rt-PA administrated within 2 h	Similar efficacy, bleedings and mortality were seen in both groups. A high number of adverse events happened.
Sors H *et al.*, 1994 (ref: 23)	RCT	53	Massive PTE (Hemodynamically unstable)	Compare reduced bolus dose of RTPA, versus 100 mg of RTPA	-	50 mg infused over 15 min and 100 mg RTPA infused within 2 h	The reduced bolus dose of RTPA showed similar efficacy versus 100 mg RTPA.
Goldhaber SZ *et al.*, 1993 (ref: 20)	RCT	101	Hemodynamically stable	Compare 100 mg of RTPA versus anticoagulant alone (heparin)	14 days	100 mg of RTPA infused within 2 h	The RTPA group showed lower rate of mortality and recurrent PE and medical embolectomy
Dalla Volta S *et al.*, 1992 (ref: 21)	RCT	20	Hemodynamically stable ^i^	Compare 100 mg of RTPA versus anticoagulant alone (heparin)	-	100 mg infused as bolus of 10 mg, followed by 90 mg over 2 h	The RTPA group showed high bleeding rate and faster and greater improvement in angiographic and hemodynamic parameters.
PIOPED Investigator, 1990 (ref: 18)	RCT	13	Hemodynamically stable	Compare 40 to 80 mg of RTPA versus anticoagulant alone (heparin)	7 days	The RTPA was infused at a rate of 1 mg/min.	The RTPA group showed a high bleeding rate and greater improvement in hemodynamic parameters but not in angiographic variables.
Levine M *et al.*, 1990 (ref: 10)	RCT	58	Hemodynamically stable	Compare a dose of 0.6 mg/kg RTPA versus anticoagulant alone (heparin)	10 days	RTPA administrated as a 2-minute infusion of RTPA at a dose of 0.6 mg/kg	The RTPA group showed greater improvement in perfusion defect with high rate of minor bleeding.

^a^pulmonary embolism,^b^tissue-type plasminogen activator, ^c^Massive PTE was determined as acute PTE with persistent hypotension (systolic pressure <90 mmHg or a decrease in systolic pressure ≥40 mmHg from baseline for at least 15 min) and cardiogenic shock (including altered mentation, oliguria or cool and mottled extremities). Submassive PTE was determined as acute PTE without systemic blood pressure reduction (systolic pressure ≥90 mmHg) with evidence of right ventricular (RV) impairment (*i.e*.: manifested by RV dilation or RV systolic dysfunction confirmed by echocardiography, RV dilation detected on computerized tomography (CT), elevated Brain natriuretic peptide (BNP) level, elevated N-terminal pro-BNP or electrocardiographic changes) or myocardial damages ( manifested by elevated troponin I and T. ^d^randomized controlled clinical trial. ^e^Moderate” PE was determined with clinical presentation of PTE along with computed tomographic pulmonary angiographic involvement of more than 70% involvement of thrombus in 2 lobar or left or right main pulmonary arteries or by a high probability of ventilation/perfusion mismatch in 2 lobes. ^f^Acute submissive PE was determined as a manifestation of a filling defect beyond the pulmonary subsegment level, normotensive, with the presence of right ventricular dysfunction on echocardiography. ^g^Hemodynamically massive PE was determined as the hemodynamic unstable or shock, or massive pulmonary artery involvement (obstructions ≥ 2lobes on CTPA or perfusion defects in ≥ 7 segments on V/Q scan) along with RVD and pulmonary hypertension on echocardiography. ^h^Intermediate PTE defined as the normotensive, hemodynamic stable PTE with the presence of right ventricular dysfunction. ^i^Hemodynamic stable patient with acute PE confirmatory confirmed with pulmonary angiography indicating vascular obstruction >30% and equivalent to a Miller index score more than 11.

**Table 2:** Sensitivity analysis for estimation of relative risks in randomized controlled trials and observational cohort studies

	**RCTs**		**Cohorts**	
	**n**	**Pooled RR (CI95%)**	**n**	**Pooled RR (CI95%)**
Total Bleeding				
Alteplase standard *vs*. low dose	3	1.91 (1.16 – 3.15)	2	0.96 (0.51 – 1.81)
Alteplase standard dose *vs*. heparin	3	1.11 (0.31 – 3.99)	1	21 (1.22 – 362.73)^*^
Alteplase low dose *vs*. heparin	2	5.69 (0.83–39.27)	1	3.61 (0.43 – 30.05)^*^
Major Bleeding				
Alteplase standard *vs*. low dose	3	1.92 (0.66 – 5.59)	2	1.04 (0.49 – 2.19)
Alteplase standard dose *vs*. heparin	3	0.60 (0.19 – 1.87)	1	7 (0.34 – 143.95)^*^
Alteplase low dose *vs*. heparin	1	1.50 (0.07–30.59)^*^	0	-
Minor Bleeding				
Alteplase standard *vs*. low dose	3	1.91 (0.68 – 5.34)	1	0.72 (0.22 – 2.29)^*^
Alteplase standard dose *vs*. heparin	3	0.50 (0.17 – 1.49)	1	15.40 (0.86–274.79)^*^
Alteplase low dose *vs*. heparin	1	1.50 (0.07–30.59)^*^	1	3.61 (0.43–30.05)^*^
Recurrence of PTE				
Alteplase standard *vs*. low dose	2	2.25 (0.51 -9.98)	1	4.10 (0.55 – 30.77)^*^
Alteplase standard dose *vs*. heparin	1	1.17 (0.30 – 4.57)^*^	1	1.44 (0.13 – 15.53)^*^
Alteplase low dose *vs*. heparin	2	0.44 (0.08 – 2.52)	0	-
Mortality				
Alteplase standard *vs*. low dose	2	1.13 (0.30 – 4.29)	2	1.15 (0.87 – 1.53)
Alteplase standard dose *vs*. heparin	3	1.08 (0.37 – 3.18)	1	0.13 (0.02 – 0.97)^*^
Alteplase low dose *vs*. heparin	3	0.45 (0.13 – 1.63)	0	-

RCT: randomized controlled clinical trial; n: number of studies within each sub-group; RR: relative risk; CI: confidence interval; PTE: pulmonary

Thrombo-emboli. ^*^no pooled estimation result.

## Conclusion

The present study concluded that in the absence of the benefit of standard-dose RTPA in comparison with dose reduction, reduced dose RTPA showed lower rates of total bleeding events and showed similar efficacy regarding mortality and PE recurrence rates compared to a standard dose of RTPA.

Moreover, compared to an anticoagulant, half-dose RTPA did not increase the bleeding, adverse effects. The study found that bleeding complications of RTPA was dependent on a dose, so owing to the minimizing risk of thrombolytic therapy, the dose reduction strategy showed a favorable risk-benefit ratio in patients with acute PE. It should be pointed out that further analysis based on cardiovascular and long-term outcomes is warranted to identify probable benefits from reduced-dose thrombolytic therapy with RTPA.

## Limitations

The present study had several potential limitations. First, despite including more than ten times as many patients as previous studies, the results of PVR, PAP, and RV dilatation or biomarker changes were not analyzed. Obviously, the quality of meta-analyses and systematic reviews are depended on how the included studies were well performed. Although the quality of the greater number of studies evaluated in this paper was noted as good, the lack of reporting morbidity outcomes was caused by limiting the generalization of conclusions about the benefit of reduced-dose RTPA. Second, non-English publications were not included. However, comprehensive literature searching detected well-designed and high-quality studies.

## Funding

This study was supported by the Research Center for Rational Use of Drugs, Tehran University of Medical Sciences (98-01-156-42133).
